# Soft Tissue Reconstruction following Hemipelvectomy: Eight-Year Experience and Literature Review

**DOI:** 10.1100/2012/702904

**Published:** 2012-05-02

**Authors:** A. Z. Mat Saad, A. S. Halim, W. I. Faisham, W. S. Azman, W. Zulmi

**Affiliations:** ^1^Reconstructive Sciences Department, School of Medical Sciences, Universiti Sains Malaysia, Kubang Kerian, Kelantan 16150, Malaysia; ^2^Orthopedics Oncology and Reconstruction Unit, School of Medical Sciences, Universiti Sains Malaysia, Kubang Kerian, Kelantan 16150, Malaysia

## Abstract

*Background and Objectives*. Hemipelvectomy is a major surgical procedure that associates with significant morbidity, functional impairment, and psychological and body image problem. Reconstruction of the defect is a challenged since a large amount of composite tissues are needed. We would like to share our eight-year experience with massive pelvic resection and reconstruction. *Methods*. A retrospective analysis of all cases of hemipelvectomy was conducted in our institution over eight-year period with particular attention given to the reconstruction choices and associated complications. *Results*. Thirteen patients were included with median age of 39 years (range 13–78) of which all had advanced tumour with stage IIb (54%) and Stage III (46%). External hemipelvectomy was performed in all cases, and resultant defects were reconstructed with variety type of flaps. These include fillet thigh flaps, regional pedicle flaps of different designs, and free flap. *Conclusions*. Massive pelvic tumour is rarely encountered in our population but can be seen across all age groups and usually due to late presentation. The defects should be reconstructed using local or regional flaps, incorporating the muscle component to enhance flap perfusion. The tissue should be harvested from the amputated limb, as it can limit the donor site morbidity.

## 1. Introduction

The surgical treatment of pelvic bone tumours is a challenge for both the oncology and reconstructive surgeon alike. External hemipelvectomy refers to the amputation of the innominate bone and is considered as one of the most invasive and destructive surgical procedure in the current period.

The decision to perform hemipelvectomy is made when partial pelvic resection does not allow for safe surgical margin or results in a functionless limb. Even though hemipelvectomy has devastating results, it provides the possibility of surgical cure or palliation for pelvic malignancies that otherwise could not be excised with preservation of the limb.

Massive pelvic resection may result in significant morbidity or functional impairment after surgery, depending on the size, location, and composite tissue loss. The defect may be large, especially in recurrence and big tumours. Reliable flap coverage is important to prevent abdominal organ herniation or exposure of bony structure and implants. Therefore, the oncologic and reconstructive surgeons must work hand in hand in the management of pelvic tumour to minimise morbidity. The purpose of this paper is to discuss the indications, the flap options for soft-tissue reconstruction, and the functional outcomes of massive pelvic resections in our institution.

## 2. Methods

Retrospective review was carried out on patients who had major pelvic resection in our institution. Over an eight year period between January, 2000 and 2008, thirteen patients with pelvic malignancy underwent hemipelvectomy. The clinical and radiological records of all the patients were reviewed. The patients were staged according to Enneking's Staging System for musculoskeletal sarcoma [1]. Hemipelvectomy procedures were also classified according to Ennekings type of pelvic resection [2]. The type of flap and its vascular pedicle supply is described in detail. Early and delayed surgical complications are highlighted and the final flap survival mentioned. The outcome of treatment in terms of flaps survival and failure were recorded. Functional outcome and major complications were also presented.

## 3. Results

Over the eight-year period, there were thirteen external hemipelvectomy performed. The average age of the patient was 39 years, ranging from 13 to 78 years with a median age of 31 years. There was an equal male to female ratio. Forty six percent were bony malignancies and 53% were soft-tissue malignancies (see Table 1). The tumours were staged using Enneking system of surgical staging of bone and soft tissue tumours at presentation [1]. All patients in our series presented with advanced disease, with stage IIB (54%) and stage III (46%). Each was high-grade, locally advanced tumour, and almost half had distant metastases.

External hemipelvectomy was indicated in all patients, because there was involvement of three or more of the major structures mentioned below. These structures are the sciatic nerve, the hip joint, the femoral neurovascular bundles, the external iliac vessels, and the bulk of gluteus muscle. In one patient, palliative surgery was offered for massive bleeding from mal-odourous, fungating tumour mass. Limb-preserving surgeries were impossible from oncological perspective or would have rendered the limb functionless in all cases.

Nine patients had pelvic resection type I, II, and III, three had extended hemipelvectomy with partial sacral resection, type I-S, II, and III, and one patient had type II and III pelvic resections according to Enneking and Dunham classification of pelvic resections [2].

As the affected limb was amputated in all cases, no pelvic bone reconstruction was necessary. However, the soft-tissue defect and coverage needed to be addressed. In the simple case where the immediate soft tissue anteriorly and posteriorly were not affected and no previous radiation, random pattern flaps can be designed to close the pelvic defect. This was possible in three of our cases. Unfortunately, they were complicated with wound infections and dehiscence in two third of the cases.

**Figure 1:**
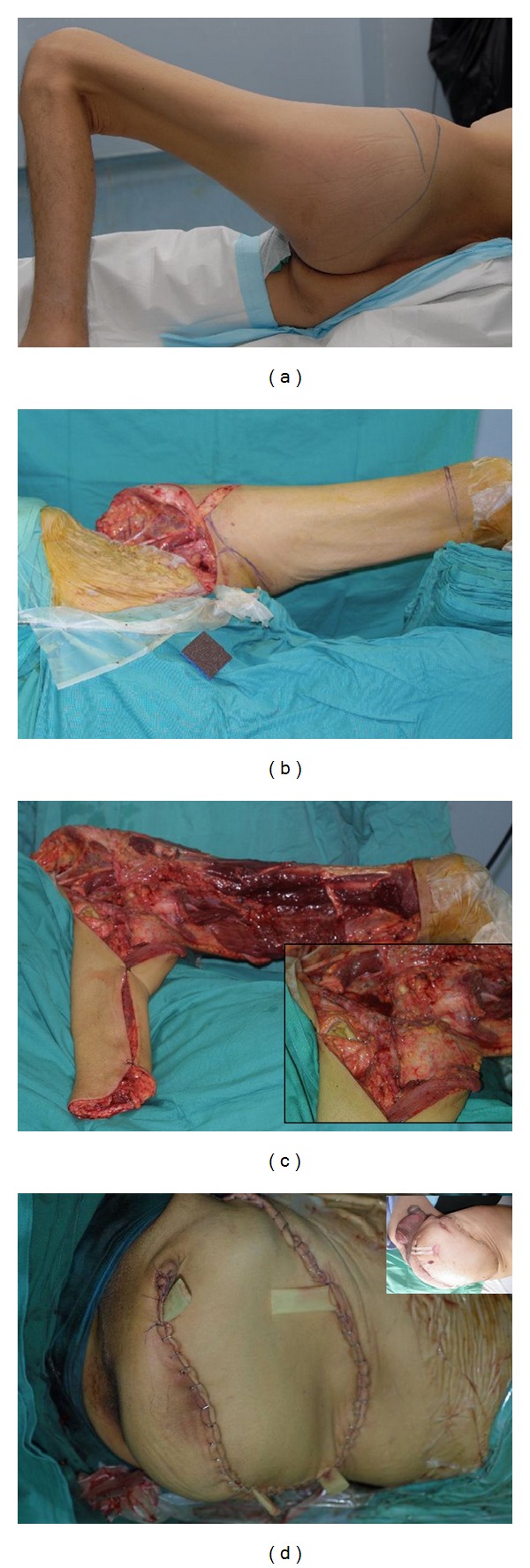
49-year-old man with late presentation of osteosarcoma involving ilium and ischium. He had hemipelvectomy and reconstruction with anteromedial thigh fillet flap. (a) Preoperative photo showing large swelling over the left hip and gluteal area. (b) Intraoperative picture showing the proposed area for fillet thigh flap to be harvested. (c) Flap was completely raised including adductor muscle group, gracilis, and sartorius. Left attached by the skin (proximal anteromedial area of groin) and its main vascular pedicle. (d) Early after procedure (Inset—Day 10 post op.).

In four cases, various types of fillet thigh flaps were designed according to the patient condition and availability of tissue for reconstruction (Table 2). In the first two patients (patient no. 1 and 2), the fillet flap was designed on the anteromedial aspect of the thigh based on the musculocutaneous perforators through the adductors, gracilis and sartorius muscles with a small skin pedicle proximally at the medial aspect of the groin. Both flaps survived completely with minor wound dehiscence which was managed conservatively (Figure 1). The other two patients (patient no 9 and 11) the fillet flap was designed with skin island. One was based on the superficial femoral vessels relying on the musculocutaneous perforators as described in the earlier group. However, the flap vein needed to be anastomosed due to injury during tumour resection. No flap related complications were seen in this patient. In the other patient, the flap was based on same vessels but relying on the perforators through the vastus muscles and rectus femoris. This patient had extended surgical procedures (pelvic exenteration) and external hemipelvectomy because of advanced disease invading the bladder, prostate, perineum, and rectum. Using multidisciplinary approached, he had external hemipelvectomy, bladder, prostate and rectal resections, creation of end colostomy, ileal conduit for urinary diversion, and soft-tissue reconstruction. He had a turbulent recovery period with multiple problems which resulted in multiple visits to operating theatre. Flap-related complications include congestion secondary to pedicle compression due to organ herniation which was relieved by exploration, wound dehiscence and infection, and late partial flap necrosis (after seven weeks of initial surgery). This was further complicated with haematoma, faecal leak, and formation of enterocutaneous fistula which prompt re-explorations. Later, he developed overwhelming sepsis and finally succumbed to the stress induced by the sepsis and multiorgan failure.

**Figure 2:**
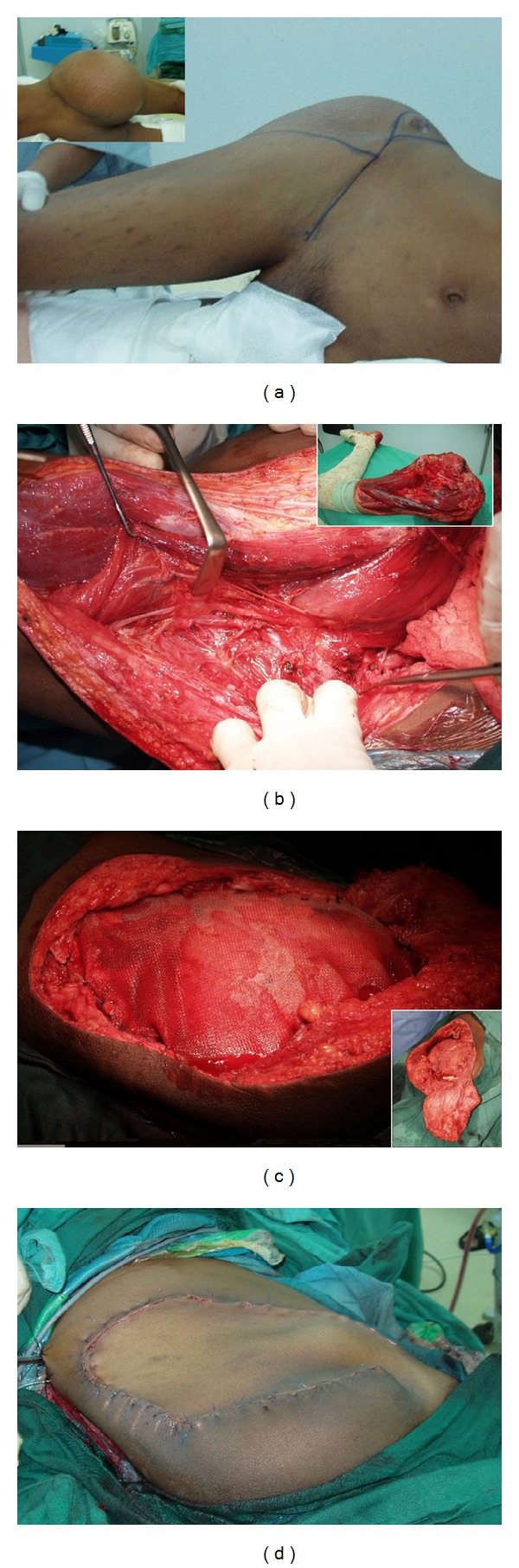
19-year-old man with a diagnosis of peripheral primitive neuroectodermal sarcoma. He had right hemipelvectomy and reconstruction with mesh and anteromedial fasciocutaneous flap based on perforators from superficial femoral vessels. (a) Pre-op photo of the large swelling over the hip and gluteal areas. (b) Dissection of the pedicle showing the intact cutaneous perforators to the skin. Inset-amputated limb showing area where the flap has been raised. (c) Peritoneal cavity is supported with mesh. Inset shows defect and the flap. (d) Immediate post op photo showing the flap inset.

Two patients had anteromedial and anterolateral thigh perforator flap which were based on the superficial femoral and the lateral circumflex femoral vessels, respectively. The latter was raised as an islanded flap and had its skin perforators dissected and traced back to the main vessel. This patient suffered from flap congestion secondary to compression from a haematoma, developed from femoral artery injury proximally. The problem resolved upon exploration and revascularisation of the flap proximal to the injured vessel. The former patient has no flap-related complication (Figure 2).

The last four patients had the traditionally described posterior thigh hemipelvectomy flap incorporating the gluteus maximus muscle. One of them had a partial flap necrosis in which the internal iliac vessel was ligated. She also had severe septicaemic shock requiring multiple inotropes which may also have contributed to her flap failure. Meanwhile, the other one had the flap modified to be a gluteus maximus myocutaneous free flap on the basis of the inferior gluteal vessels, because the vessels had to be divided as origin from the internal iliac were encased with the tumour. The flap survived well apart from transient congestion due to haematoma formation around the pedicle that was surgically removed.

At the time of review, eight patients were still alive since surgery with a median survival of 5 months, ranging from 2 months to 9 months. Five patients died since the operation, with a median survival of three months with the survival period ranges from six days to eighteen months after surgery.

In total, there are six patients who ambulate with crutches, one with a walking frame, one with a wheelchair, and four are bed bound after the surgery. Those who were bed bound had poor rehabilitation and died early, within three months of surgery. None of the patients had a prosthesis fitted for ambulation in our series. Generally, all patients have satisfactory wound coverage and acceptable external appearances.

## 4. Discussion

Pelvic resection of the innominate bones and or sacrum is a rare procedure, usually indicated for tumours, severe infections, or sometimes after trauma. Traditionally, hemipelvectomy will be the only viable option, but with the advent of modern and sophisticated radiological tools, better imaging techniques provide means for less invasive surgery without compromising the outcome or the safety margin. This would mean that limb sparing surgery would be an option if the vital structures were not involved [3–5].

The decision for external hemipelvectomy is often difficult. Many factors need to be considered such as functionality, body image, emotional acceptance, and, above all, tumour clearance. If limb sparing does not allow for safe margin, or will render the limb functionless, external hemipelvectomy should strongly be considered.

Billroth first attempted a hemipelvectomy in 1891. Unfortunately, the patient died within several hours of the operation. Girard of Switzerland was the first to successfully achieve this endeavour in 1895 [6, 7]. Since then, operative mortality has fallen from an initial high of 60% to less than 5% today [8]. An English surgeon, Hogarth Pringle, established the essential technique for hemipelvectomy in 1916. Since that time, there have been many advances in flap technique and choices [1, 6–17].

Enneking and Dunham proposed a classification scheme for description of various subtype of pelvic resections [2]. Type I resections denote those that involve all or part of the ilium, sparing the acetabulum. The extension of type I resection to include part of the sacrum or gluteal muscle are called “type I-S” and “type I-A” respectively. Type II resections are those involving periacetabular region and frequently involving the femoral head (Type II-A). Type III resection involves the ischiopubic region of the pelvis (i.e., medial to the acetabulum and lateral to symphysis pubis). Partial or complete sacral resection on its own is referred to as type IV pelvic resection.

For extensive pelvic resection such as external hemipelvectomy, where amputation is considered, several options are available for reconstruction. Depending on the location of the tumour or local extension of the tumour, the traditionally described posterior flap and anterior flap hemipelvectomy can be utilised [3]. The inclusion of a muscular component (gluteal muscles and anterior thigh compartment muscles, resp.) into these flaps has decreased the incidence of complications associated with the flaps, such as necrosis and flap failure [3].

The posterior flap hemipelvectomy relies on the inferior and superior gluteal arteries which arise from the internal iliac artery. However, in some cases, the internal iliac vessel may need to be sacrificed if it is involved. In this case, the posterior thigh flap may still be used depending on the blood supply to the gluteus maximus muscle at its sacral origin, or it can be raised as free flap like in one of our cases. The incidence of posterior flap failure is slightly more in those with the common iliac artery ligated, compared to ligation of vessels at the external iliac, but they are not statistically significant [3].

The anterior flap hemipelvectomy is generally used as an alternative to a posterior flap, when the tumour is located within and around the posterior aspect of the pelvic region which precludes it use. The anterior flap is based on the superficial femoral vessels, which is a continuation from the external iliac artery. As with the posterior flap, the inclusion of the muscles from the anterior compartment of the thigh improved the perfusion to the flap and minimised the risk of flap failure [3, 5].

A high flap complication rate was seen in the posterior subcutaneous skin flap. The complication rate was 80% with 55% necrosis as reported by Douglass et al. [9]. After flap modification was done to include the gluteus maximus muscle, there was no flap necrosis as reported by Karakousis and Vezeridis et al. [5] Frey et al. [18] first described the anterior flap incorporating rectus femoris and vastus intermedius muscles as a myocutaneous skin flap (quoted by Apffelstaedt et al.) [3]. Karakousis later documented that the anterior flap without the muscle is associated with a high failure rate which can be rectified by incorporating the muscle as described earlier [3, 5].

In certain cases, a local option of simple random pattern flap may be used safely by properly designing the skin flap and by respecting the perforators encountered whilst raising the flaps. This has been used in several cases in our series without any major complications.

Sometimes, the defect can be massive and larger soft tissue coverage is needed. For patients undergoing external hemipelvectomy or hindquarter amputation, the soft tissue can be recovered from the amputated limb. Fillet thigh flap either free or pedicle and free fillet lower leg flap has been described before. Yamamoto et al. described a case series of free fillet lower leg flap with its surgical approach [19] and later published an improvised technique together with the long-term results [10].

Fillet thigh flap can be harvested as a pedicle flap when amputation of the limb has been considered. This is based on the “spare part” concept where the residual tissue from the amputated limb can be utilised and thus eliminating donor site morbidity of healthy tissue. When hemipelvectomy is considered, this flap can be based on the superficial femoral artery, which is the main blood supply to the muscles and skin at the anteromedial thigh area. The flap can be designed as a pedicle, an islanded or free flap [20] and a large flap can be harvested from the whole leg as described by Küntscher et al. in their series [20], where 75 × 30 cm and 71 × 22 cm flaps were raised to cover pelvic defect in two patients. The complications rate in his series in the limb fillet flap was 19% of which only one related to flap survival (partial necrosis) and the rest were wound infections and fistula formations.

**Figure 3:**
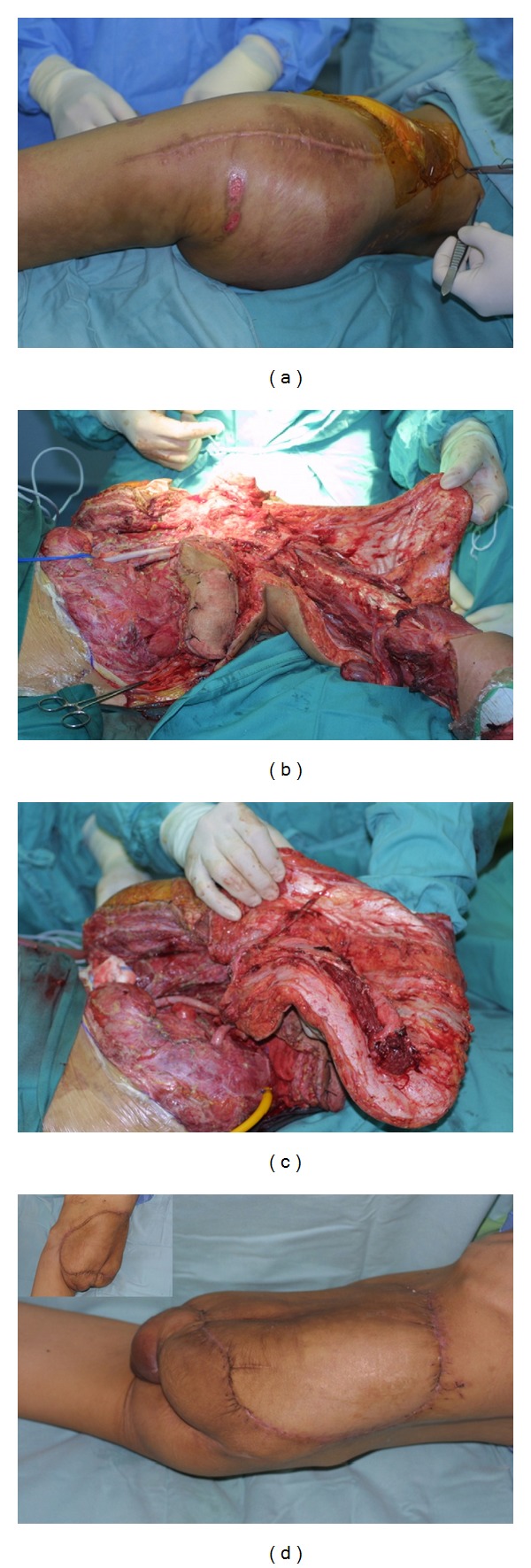
Case of 24-year-old lady with recurrent osteosarcoma previously treated with limb sparing surgery and modified hip arthroplasty, completed radiotherapy and chemotherapy. She had recurrent eight months later and external hemipelvectomy was done. (a) Preprocedure showing large swelling left buttock area. (b) The fasciocutaneous flap over the anterolateral thigh is raised on its pedicle-lateral circumflex femoral vessels. (c) Flap has been completely raised on its pedicle and inset. (d) Lateral and anteroposterior view one month post op.

In one of our case (patient no. 8—Figure 3), the concept of fillet thigh flap was used with slight modification, a large skin island was raised on the basis of perforators arising from the lateral circumflex femoral artery utilising skin over the anterolateral aspect of the thigh. Initially, it was raised as a pedicle flap but later needed to be revascularised as a free flap due to proximal injury of the femoral artery causing haematoma and flap congestion. There were no other flap related complications afterward and the patient has a good outcome.

The vertical rectus abdominis myocutaneous (VRAM) flap has been described before for soft-tissue pelvic reconstruction and deemed to be highly reliable with a lower flap complication rate [11, 21–23]. However, in the case of major pelvic resection, the use of ipsilateral inferiorly based VRAM was associated with high complication rates (62.5%) [21]. This may be due to dissection close to its pedicle which increases the risk of injury or compression to the vessels. The use of contralateral VRAM flap in the series showed much lower flap-related complications [21]. Buchel et al. published a large series of VRAM used in pelvic reconstruction (perineal area), where this shows that the flap is reliable, with minimal flap related complications and donor site morbidity [24].

The gluteus maximus musculocutaneous flap is well described and shows a reliable vascular supply on the basis of the gluteal arteries. The new version of this flap includes the fasciocutaneous flap via its perforators. In pelvic reconstruction, the bulk of the muscle is often needed which leaves the traditional musculocutaneous type being a preferred choice. One should keep this in mind if considering using of this flap when the internal iliac vessels are sacrificed, as perfusion may be compromised [3]. There are couple of ways to improve flap survival, either raise it as free flap as in our case, or preserving the sacral origin of the gluteus maximus.

“Reverse” Latissimus Dorsi myocutaneous flap was used by Muramatsu et al. to cover and fill up the dead space in the posterior aspect of the pelvis [23]. This is based on the secondary blood supply to the latissimus dorsi muscle, of which the presence and location was reported in an anatomical study by Stevenson et al. [25]. There are three large perforators originating from ninth, tenth, and eleventh intercostal vessels located five centimetres from the midline [25]. The limitation of this reverse flap is that of its reach and the size of the skin paddle.

## 5. Conclusions

Massive pelvic resection is a procedure with considerable morbidity and poses challenges for the oncology and reconstructive surgeons. Careful assessment and appropriate imaging modalities should be utilized as part of combined preoperative planning among the surgeons. On the basis of our experiences and previous literatures, these procedures associated with a lot of morbidity and complications. The best option for reconstructive the defect should be concentrated on utilizing the tissue from the amputated part if at all possible to minimize donor morbidity. As we have demonstrated, multiple options are available for raising the flaps. It could be pedicled or free flap, the skin could either be attached or islanded, and the muscle could be incorporated or could be left for it to be based on the skin perforators alone. Thorough discussions with the patient and close relatives are needed as this will impact their way of life, body image, and psychology. External hemipelvectomy may offer a chance of palliation and possibly cure when lesser surgical options have been exhausted.

## Figures and Tables

**Table 1:** Patient summary.

No.	Age/ sex	Diagnosis	Stage	Resection type^t^	Reconstruction	Complication	Chemotherapy/ radiotherapy	Outcome
1	49/M	Osteosarcoma	Stage IIB	P I-A, II, III	Musculocutaneous anteromedial fillet thigh flap	Small wound dehiscence	No	Alive 13 months post op, no local recurrent, crutches
2	25/M	Neurofibrosarcoma	Stage IIB	P I-S, II, III	Musculocutaneous fillet thigh flap SFA pedicle	Small wound dehiscence, dura tear intra-op	No	Died 2 months post op, bedbound.
3	19/M	Peripheral primitive neuroectodermal sarcoma	Stage III	P I-A, II, III	Anteromedial fasciocutaneous	No	Radiotherapy	Died 18 months post op, local recurrent and metastasis walking with crutches and riding motorbike, enjoy his life
4	54/F	Metastatic adenocarcinoma unknown primary	Stage III	P II, III	Random pattern	Wound infection, phantom limb	No	Died 2 months post op at home, bedbound, low self esteem
5	78/M	Malignant fibrous histiosarcoma	Stage III	P I-A, II, III	Random pattern	Septicaemia, wound infection	No	Uncontactable
6	69/F	Liposarcoma	Stage IIB	P II, III	Random pattern	Bladder dysfunction	Radiotherapy	Alive 14 months post op, no recurrent. walking frame
7	54/F	Metastatic SCC	Stage III	P I, II, III	Posterior thigh—gluteal myocutaneous flap—internal iliac vessel ligated	Died, partial flap necrosis	No	Died 6 days post op
8	24/F	Osteosarcoma (recurrent)	Stage IIB	P I, II, III	Fasciocutaneous skin perforators based on Lat circumflex femoral	Flap congestion 2ndary to femoral artery injury/ haematoma, re-explore x2 revascularisation of flap prox. to injured area	Yes	Alive 17 months post op, no recurrent, crutches
9	31/F	Synovial sarcoma	Stage III	L hemipelvectomy P-Is, PIIa, PIII	Musculocutaneous fillet thigh flap with vein anastomosis	Pain, phantom limb	Radiotherapy	Alive 14 months post op, wheelchair
10	17/M	Osteosarcoma	Stage IIB	P I, II, III	Musculocutaneous posterior flap hemipelvectomy	No	Yes	Alive 12 months post op, wheelchair
11	48/M	Chondrosarcoma (recurrent)	Stage IIB	P-I, II, III, plus pelvic exenteration, perineal resection, ileal conduit, colostomy	Musculocutaneous anterior fillet thigh flap	Flap congestion (compressed by bowel), wound dehiscence/Infection, haematoma from bleeder of int. iliac vessel, central line sepsis, enterocutaneous fistula, and delayed partial flap necrosis after multiple exploration of wound/surgical sites	No	Died 3 months post op, bedbound
12	13/M	Osteosarcoma	Stage III	P I, II, III	Gluteus maximus myocutaneous free flap	Flap congestion secondary to haematoma	Chemotherapy	Alive 10 months post op, crutches
13	18/M	Osteosarcoma	Stage III	P I, II, III	Posterior thigh—gluteal myocutaneous flap	No	Chemotherapy	Alive 6 months post op, crutches

^t^ According, Enneking and Dunham classification. See text for full description.

**Table 2:** Type of flap used and their related complications.

Flap design	Type	Pedicle	Flap complications	Number of cases	Subtotal
Fillet thigh	Musculocutaneous	SFA	Minor wound dehiscence. Managed conservatively	2	
	Islanded musculocutaneous	SFA	Early congestion-pedicle compressed by bowel-resolved after exploration Later partial flap necrosis after multiple wound re-exploration secondary to haematoma/fecal leakage and enterocutaneous fistula	1	4
		SFA (with venous anastomosis)	None	1	

Anterolateral thigh	Islanded fasciocutaneous	Lateral circumflex femoral	Flap congestion due to proximal femoral artery injury and haematoma formation. Resolved after reexploration and revascularization of flap proximal to injury area	1	1

Anteromedial thigh	Fasciocutaneous	SFA	None	1	1

Posterior thigh	Musculocutaneous (gluteus maximus)	Internal iliac preserved	None	2	3
		Internal Iliac ligated	Partial loss, patient was on multiple inotropes secondary to shock and sepsis. Died day 6 post op	1	

Free flap	Gluteus maximus myocutaneous free flap	Inferior gluteal artery and vena comitans	Transient congestion secondary to hematoma which was surgically evacuated	1	1

Random pattern	Fasciocutaneous		None	1	3
			Wound infections and dehiscences manage conservatively	2	

			Grand total: 13		

SFA = Superficial femoral artery.
